# Respiratory symptoms, fractional exhaled nitric oxide & endotoxin exposure among female flower farm workers in Ethiopia

**DOI:** 10.1186/s12995-015-0053-x

**Published:** 2015-02-26

**Authors:** Amare W Nigatu, Magne Bråtveit, Wakgari Deressa, Bente E Moen

**Affiliations:** Department of Global Public Health and Primary Care, University of Bergen, Bergen, Norway; School of Public Health, Addis Ababa University, Addis Ababa, Ethiopia

**Keywords:** Endotoxin exposure, Respiratory symptoms, Fractional exhaled nitric oxide, Greenhouse, Flower farm

## Abstract

**Background:**

Greenhouse workers are exposed to organic dusts, and they are thereby at risk of developing airway disorders. This study aims to measure personal endotoxin exposure, assess respiratory symptoms and measure fractional exhaled nitric oxide (FeNO) among female flower farm workers in Ethiopia.

**Methods:**

A cross-sectional study involving female workers (n = 248) from four flower farms was conducted. The workers were interviewed for respiratory symptoms using a standard questionnaire. Workers from two of these farms also participated in personal endotoxin sampling (46 workers, 75 measurements) on glass fiber filters (0.2 μm pore size) inside conductive 25 mm Millipore cassettes for sampling of the “total dust” fraction. They also participated in FeNO (n = 114) measurements with a portable electrochemistry-based sensor. Chi-square and independent t-tests were used to compare categorical and continuous variables respectively. A mixed-effects model was used to analyze exposure determinants.

**Results:**

Endotoxin exposure had a geometric mean (GM) of 22.8 endotoxin units (EU)/m^3^ with a maximum of 180 EU/m^3^. Greenhouse workers had significantly higher endotoxin exposure than workers outside the greenhouses (GM = 26.7 vs. 19.3 EU/m^3^ respectively; p < 0.05). The mean age of the workers was 24 years, and their mean working time in the flower farm was 21 months. Greenhouse workers had higher prevalence of self-reported respiratory symptoms than those outside greenhouses. However, after adjusting for education only blocked nose remained significant. The FeNO concentration ranged 5–166 ppb (GM = 14 ppb). Two workers had FeNO concentration above 50 ppb. FeNO levels differs significantly between the farms but there was no difference between workers inside and outside greenhouses.

**Conclusion:**

Greenhouse workers at flower farms had higher prevalence of blocked nose than workers outside, which may indicate the presence of rhinitis. Endotoxin exposure was low. There were few workers with objective signs of airway inflammation; this might be because the mean working time in the greenhouses was only two years. We suggest further studies to evaluate the effect of longer employment and exposure time as well as to investigate possible exposure to pesticides and other components in the bio-aerosols.

## Background

The cultivation of roses inside greenhouses is a rapidly growing economic activity in Ethiopia, and covers 120 hectares of land with over 50,000 workers [1]. The greenhouses are characterized by elevated temprature, humidity and poor ventilation [2-4]. Pesticides of different types, chemical fertilizers and to some extent biological agents are widely used to enhance the growth of flowers [5].

It is likely that people in the agriculture sector are exposed to organic dusts during their daily routines [6-8]. Part of the agriculture process involves work in greenhouses, including cultivation of flowers and vegetables. Because of the enclosed space and poor ventilation, greenhouse work might also be associated with high exposure levels to organic dust [9]. Several studies among greenhouse workers have shown high prevalence of respiratory symptoms such as chronic cough, dyspnea, chest tightness and rhinitis, as compared to controls [2,10-12]. In 2012 we conducted a study among flower farm workers in Ethiopia and found that workers inside the greenhouse had significantly higher self-reported prevalence of chronic respiratory symptoms compared to workers outside the greenhouse as well as compared to a control group of supermarket workers [13]. No objective measurements of occupational exposure or respiratory health were performed in that study.

The organic dust in greenhouses has many components such as fungal spores, bacteria, and endotoxins [4,14,15]. Endotoxins are a cell-wall component of gram-negative bacteria, and can be inhaled by workers [16,17]. It is suggested that they are involved in the development of respiratory disorders such as obstructive lung disease [17]. As many of the Ethiopian farm workers had respiratory symptoms, which can be considered typical for airway obstruction [9,13], we decided to measure endotoxin exposure levels in their work environment.

Adverse respiratory effects due to organic dust exposure might be associated with eosinophilic inflammation and increased fractional exhaled nitric oxide (FeNO) among workers [18]. The measurement of FeNO involves a simple and non-invasive method. The technique uses a portable device. This gives fast and reliable results in the field [19]. Occupational exposure to organic dust and measurement of FeNO in the agricultural sector has been used in the Western countries to study several work settings [20]. However, few of these studies are conducted among greenhouse workers [4,11]. Moreover, there is no information about endotoxin exposure and FeNO among flower farm workers in Ethiopia.

The objective of the present study was to evaluate the exposure to endotoxins; to assess the prevalence of respiratory symptoms and to measure FeNO among female workers inside and outside greenhouses in flower farms in Ethiopia.

## Methods and materials

### Study design and study settings

A cross-sectional study was conducted from July to October 2013 in four flower farms in the three main geographical areas for cultivation of roses in Ethiopia (Hollota/Addis-Alem, Debrezeit and Ziway areas). Due to practical reasons such as location and accessibility, we used convenient sampling to select the farms. The workers in each of the farms were divided into two observational groups based on their workstation (inside vs. outside greenhouses) for an interview, FeNO measurement and endotoxin sampling under their normal working conditions. All the four farms were included in the assessment of respiratory symptoms, but due to limited resources only two farms were included in the measurements of endotoxin and FeNO. The farms were visited before the actual data collection and the farm managements were informed about the purpose of the study.

The farms only grow roses, and vary in size from 5–51 hectares, while the total number of workers ranged from 350 to 1,300 (Table 1). The production rate is relatively constant, but at times there is peak production to meet increased demands. All the farms use chemical fertilizers and pesticides to enhance the growth of flowers and to control pests. In addition, Farm I also uses mites as biological control, however, we did not collected information on the types of mites used. The pesticides are manually mixed at a central location inside greenhouses and distributed through pipelines. The sprayers use a nozzle or sprayer gun to spray the pesticide. In all the farms, except Farm IV, spraying takes place in the morning during which time the other greenhouse workers are relocated to other greenhouses until the spraying is completed. In Farm IV spraying is done late in the afternoon after all greenhouse workers have completed their work and left the farm.

**Table 1:** **Description of the flower farms and workers participated in the study**

**Farm characteristics**	**Farm ID**			
	**I**	**II**	**III**	**IV**
Farm size (hectares)	51	12	5	41
Number of greenhouses	21	7	3	5
Number of pack-houses	2	1	1	2
Number of workers	1,300	700	350	1295
Sample Size				
Interview	67	47	41	93
FeNO measurement	67	47	NA	NA
Endotoxin measurement	25	21	NA	NA
Pest control methods	CP, BA	CP	CP	CP
Pesticide spraying time	6:00–10:00 AM	6:00–10:00 AM	6:00–10:00 AM	3:00–6:00 PM
Flower cultivated	Roses	Roses	Roses	Roses

NA: Not Applicable; CP: Chemical Pesticides; BA: Biological Agents.

The great majority of the workers are females, who either harvest and weed flowers inside greenhouses or trim and pack roses in a pack-house before storage in a cold room until transporting them to Addis Ababa for export. The few men work as pesticide sprayers and also perform other activities including repairing the greenhouses, collecting and disposing of wastes as well as transporting harvested flowers.

### Study participants

Female workers from the selected farms participated in the study. Based on the prevalence of shortness of breath (70%) among Ethiopian flower farm workers [13], a total of 248 participants (divided in two groups, i.e., inside vs. outside greenhouse) were needed to achieve 80% statistical power with significance level set to P < 0.05. The plan was to have equal number of workers from each of the four farms as well as from inside and outside greenhouses, and overall 122 and 126 workers participated from inside and outside greenhouses, respectively. The same participants from Farm I & II (n = 114) were also invited to participate in the FeNO measurements. Participants were randomly selected by the researcher from the personnel registration book in Farm II & III, while in Farm I & IV, the selection was done together with the farm administration. The principal investigator did all the interviews and the FeNO measurements in a separate room inside the farm compound. The FeNO instruction was sometimes done in groups of 2–4 workers.

### Interview

A part of the standardized questionnaire from the British Medical Research Council (BMRC) on respiratory health was used to assess chronic respiratory symptoms. The questionnaire was adapted to the Ethiopian context, and translated from English to the local language; the symptoms included cough first thing in the morning, day/night time cough, shortness of breath, chest tightness and wheezing as well as previous diseases such as bronchitis, pulmonary TB, asthma and chest injury (yes/no). In addition, questions about upper airway symptoms (runny nose, sneezing and blocked nose) were also included [21]. Moreover, previous flower farm experience (months), socio-demographic characteristics such as age, education (years in school), types of job (cutter & weeder, packing, deleafing, grading, quality control), present and previous smoking habits (yes/no) and the use of Personal Protective Equipment: gloves and respiratory protective device (yes/no) were also asked.

### FeNO measurement

FeNO measurements were taken for each person in a sitting position during the working day between 10:00 and 15:00 hours, using a portable electrochemistry-based sensor (NIOX MINO; Aerocrine AB, Solana, Sweden) in accordance with the American Thoracic Society & European Respiratory Society recommendations for online measurement of FeNO [22,23]. The mean ambient NO, room temperature and relative humidity of the room where FeNO measurement performed were registered daily; below 5 ppb, 22°C (18 – 28°C) and 70%, respectively.

### Endotoxin sampling and analysis

Full-shift personal endotoxin air samples were collected in the two selected farms (inside vs. outside greenhouses). The number of endotoxin samples was based on the Rappaport and Kupper’s recommendations for exposure studies; 10–20 measurements per observational group, two measurements of 5–10 randomly selected individuals per group [24]. Totally 46 female workers were invited for sampling; 25 from Farm I, and 21 from Farm II. Among these, 31 workers had repeated samples.

Sampling was performed from the workers’ breathing zone during the summer months, which is a rainy season in Ethiopia. Side Kick Casella Pump at a flow rate of 2 L/minute connected to 25 mm closed-face conductive Millipore cassettes containing a glass fiber filter with 0.2 μm pore size was mounted onto each worker for sampling of the “total dust” fraction. The mean sampling times were 350 (275 – 425) minutes and 358 (240 – 405) minutes for Farm I and II, respectively. The pumps were calibrated using a Rotameter every day before starting the measurement, and the air flow rate was also measured at the end of the measurements. Two samples were excluded due to a reduction in air flow of more than 10% during the sampling day. Filters were kept cold at about 4°C inside a box filled with ice bags until the samples were transported to Norway and then to Sweden for analysis at Lund University Medical Laboratory. The samples were transported as hand luggage on the flights, and were outside the cold box for a total of about 15 hours. The glass fiber filters were immersed into 0.05% Tween-20 pyrogenic free water and shaken on a rotary shaker for 1 h. Endotoxin extracts were analyzed using kinetic chromogenic Limulus amebocyte Lysate (LAL) Assay. The results were expressed as Endotoxin Unit (EU) per filter. A total of 27 samples had endotoxin values below the Limit of Quantification (LOQ), which was set to 10 EU per filter by the laboratory. In calculations of exposure levels (EU/m^3^), the amount of endotoxins on the respective filters were divided by the air volume that had passed through that filter. The measurements below LOQ were set as LOQ/2^1/2^ in further data handling. This cut-off level has been suggested when less than 50% of the data are nondetectable, and the geometric standard deviation is below 3.0 [25]. The resulting limit of detection (LOD) for the endotoxin exposure corresponding to the 27 samples < LOQ varied from 8.7 to 14.7 EU/m^3^ due to different air volumes.

### Ethics

The research proposal was submitted and approved by the Ethical committees both in Norway and Oromia Regional State Health Bureau, Ethiopia. All the participants were informed about the purpose of the study and written consent was obtained.

### Statistical analysis

The data was plotted into SPSS version 21 and analyzed using descriptive statistics. The distribution of endotoxin and FeNO data were skewed, and were therefore log-transformed in order to compare the levels between the groups. Chi-square test for categorical variables, and independent t-tests for continuous variables were used to test differences between the groups. The significance level was set to 0.05. A linear mixed-effects model was used to analyze the determinants of endotoxin exposure among the female workers, since we had repeated measurements of endotoxins on several of the workers. The individual female participants were included in the model as random effects. The farms (Farm I vs. Farm II) and workstation (inside vs. outside greenhouses) were set as fixed effects. Logistic regression was used to test differences in symptoms between groups by adjusting for education. Education was the only potential confounder among those tested that was significantly different between workers inside vs. outside greenhouses.

## Results

### Characteristics of the study participants

The workers were categorized by workstation based on whether they work inside the greenhouse or not. The response rate for the interview was 100%. The mean age of all female workers was 24 years (range 14 – 60 years). There were no differences in age, previous respiratory diseases and duration of work experience between inside and outside greenhouse workers (Table 2). Workers outside the greenhouse had significantly higher education than those working inside (Table 2). None of the female workers were current smokers or had any previous history of smoking. The majority of the workers (97%) used biofuel (wood, cow dung & charcoal) as energy source for cooking and other domestic purposes. A quarter of the participants had domestic animals, and 7% shared their living space with the animals.

**Table 2:** **Characteristics of the female flower farm workers by workstation (inside vs. outside greenhouse)**

**N**	**IGH**	**OGH**
	**122**	**126**
**Years of age**		
Mean (Range)	24 (14 – 48)	23 (16 – 60)
**Cooking energy N (%)**		
Biofuel-wood-dung-wood-charcoal	119 (98)	122 (97)
Kerosin	2 (1.6)	4 (3)
Both	1 (0.8)	0
**Educational level N (%)**		
No Education (0–4 school years)	55 (45)	25 (20)*
Education (>5 school years)	67 (55)	101 (80)
**Work experience in Months**		
Mean (range)	21 (1–96)	22 (1–96)
**Previous Agricultural experience N (%)**	28 (23)	38 (30)
Previous Agricultural experience in months Mean (SD)	20 (18)	8 (8)
**Domestic animals N (%)**	27 (22)	34 (27)
Shared room with domestic animals	7 (6)	10 (8)
**Pesticide use N (%)**		
For household pests^a^	52 (43)	60 (50)
For mosquito control	23 (19)	28 (22)
**Previous diseases N (%)**		
Chest injury/operation	1 (0.8)	0
Bronchitis	9 (7.4)	12 (9.5)
Pneumonia	3 (2.5)	4 (3.2)
Pulmonary TB	1 (0.8)	2 (1.6)
Asthma	4 (3.3)	3 (2.4)
Smoking		
Current & Previous smokers	None	None

IGH: Inside Greenhouse; OGH: Outside Greenhouse; *Pearson chi-square test, P < 0.001; ^a^Fleas, bed bugs, lice, mice.

### Endotoxin exposure measurements

The personal endotoxin exposure ranged from < LOD to 180 EU/m^3^ with a geometric mean of 22.8 EU/m^3^ (Table 3). Only one of the samples exceeded the Dutch recommended health-based exposure limit value for endotoxins in inhalable dust of 90 EU/m^3^ [26]. The endotoxin exposure inside the greenhouses was significantly higher than outside the greenhouses (p < 0.05) (Table 3). Workers in Farm I had significantly higher endotoxin exposure than workers in Farm II (GM = 29.2 Vs. 17.3 EU/m^3^; P < 0.01). The greenhouse workers in Farm I were the subgroup with the highest endotoxin exposure (GM = 37.8 EU/m^3^) (Table 3).

**Table 3:** **Personal endotoxin exposure among female flower farm workers according to farm (I or II) and workstation (inside vs. outside greenhouse)**

**Farm ID**	**Workstation**	**Nw**	**Ns**	**Ns <LOD**	**Endotoxin (EU/m3)**		
					**AM**	**Range** ^**a**^	**GM (GSD)**
Farm I	IGH	10	19	2	47.3	9.2 – 180	37.8 (2.0)*^b^
	OGH	15	20	8	28.2	10.0– 64.6	22.9 (2.0)
	Total	25	39	10	37.5	9.2 – 180	29.2 (2.1)**^c^
Farm II	IGH	12	19	8	24.9	8.7 – 60.9	18.9 (2.1)^ns,b^
	OGH	9	17	9	17.1	9.1 – 34.7	15.8 (1.5)
	Total	21	36	17	21.3	8.7 – 60.9	17.3 (1.9)
Total	IGH	22	38	10	36.1	8.7 – 180	26.7 (2.2)*^b^
	OGH	24	37	17	23.1	9.1 – 64.6	19.3 (1.8)
	Total	46	75	27	29.7	8.7 – 180	22.8 (2.1)

Independent sample t-tests were used to compare groups.

EU/m^3^: Endotoxin Units per cubic meter; Nw: Number of workers; Ns: Number of sample; <LOD: Below Limit of Detection; AM: Arithmetic Mean; GM: Geometric Mean; GSD: Geometric Standard Deviation; IGH: Inside Greenhouse; OGH: Outside Greenhouse (packhouse); **P < 0.01, *P < 0.05, ns = not significant, ^a^The lowest exposure in all subgroups are estimated values for results below LOD; ^b^Comparing samples inside and outside greenhouses; ^c^Comparing Farm I and Farm II.

The day-to-day endotoxin exposure variability (within workers) was higher than the between worker variability (Table 4). In a mixed-effects model analysis, the fixed factors workstation and farm, explained 17% of the total variance in endotoxin exposure (Table 4). The fixed factors explained parts of the between-worker variance only. Work in Farm I was associated with 1.7 times higher personal exposure to endotoxin compared to Farm II, while those working inside the greenhouse had 1.4 times higher exposure than those working outside.

**Table 4:** **Linear mixed-effects model for the log**_**e**_ **-transformed personal endotoxin exposure levels of female workers**

	**Endotoxin (EU/m** ^**3**^ **)**		
	**Random-effects**	**Mixed-effects**	**Effect (e** ^**β**^ **)**
	**Model β (SE)**	**Model β (SE)**	
Intercept	3.14 (0.09)**	2.67 (0.15)**	
Workstation (OGH = 0 & IGH = 1)		0.35 (0.16)*	1.4
Farm ID (Farm II = 0 & Farm I = 1)		0.53 (0.16)**	1.7
WWδ (SE)	0.35 (0.09)	0.35 (0.09)	
BWδ (SE)	0.17 (0.09)	0.08 (0.08)	
% of explained variance		17%	

SE: Standard Error; β: Regression Coefficient; IGH: Inside Greenhouse; OGH: Outside Greenhouse; WWδ: Within Worker Variance; BWδ: Between Worker Variance; **P < 0.01, *P < 0.05.

### Use of Personal Protective Devices (PPD)

None of the female workers in any of the four flower farms used any type of Respiratory Protective Devices (RPD). Almost all (99.6%) of the workers indicated the reason for not using RPD was because it is unavailable or not provided at the work place. Gloves at work were used by 64% of all female workers (78% and 50% among inside and outside greenhouse workers respectively) with significant differences between the farms (p < 0.01). The reported reason for not using gloves was mainly because gloves were not available or provided.

### Prevalence of chronic respiratory symptoms

Among all invited workers in the four farms shortness of breath (53%), chest tightness (27%) and morning cough (25%) were the most frequently reported symptoms (Table 5). Morning cough, runny nose and blocked nose were significantly higher among greenhouse workers compared to those working outside (Table 5). When adjusting for education by logistic regression analyses, only the prevalence of blocked nose remained significantly higher among workers inside greenhouses. The prevalence of morning cough and day/night time cough with sputum were significantly different among the four flower farms (p = 0.009 & 0.028 respectively), while the other symptoms did not differ among these groups. Farm I had the highest prevalence for most of the symptoms (results not shown). When comparing results of Farm I & II, where we also have measurements of personal endotoxin exposure and Fractional exhaled Nitric Oxide (FeNO), Farm I has relatively higher prevalence for most of the symptoms. The prevalence inside the greenhouse was higher than outside for most of the symptoms. Due to small numbers, the groups inside and outside the greenhouse in these two farms were not statistically compared after stratification by farm.

**Table 5:** **Prevalence of respiratory symptoms by workstation (inside vs. outside greenhouse)**

	**Workstation**			**Adjusted OR (95% CI)** ^**a**^
	**Total**	**IGH**	**OGH**	
	**N = 248**	**N = 122**	**N = 126**	
	**n (%)**	**n (%)**	**n(%)**	
Morning cough	63 (25)	38 (31)*	25 (20)	1.19 (0.63, 2.26)
Cough day/night time	41 (17)	23 (19)	18 (14)	1.08 (0.53, 2.21)
Cough 4–6 days a week	47 (19)	29 (24)	18 (14)	1.31 (0.66, 2.64)
Cough more days in 3 months	18 (7)	11 (9)	7 (6)	1.11 (0.39, 3.13)
Morning cough with sputum	39 (16)	22 (18)	17 (14)	1.03 (0.49, 2.14)
Cough day/night time with sputum	19 (8)	11 (9)	8 (6)	1.15 (0.43, 3.09)
Cough 4–6 days a week with sputum	25 (10)	15 (13)	10 (8)	0.99 (0.39, 2.45)
Cough more days in 3 months with sputum	11 (4)	7 (6)	4 (3)	1.01 (0.27, 3.79)
Shortness of breath walking on level ground/slight hill	131 (53)	60 (49)	71 (56)	0.65 (0.38, 1.09)
Shortness breath walking own pace	38 (15)	22 (18)	16 (13)	1.16 (0.56, 2.43)
Wheezing	40 (16)	20 (16)	20 (16)	0.69 (0.33, 1.46)
Chest tightness	66 (27)	34 (28)	32 (25)	0.89 (0.49, 1.62)
Sneezing	70 (29)	32 (26)	38 (30)	0.74 (0.42, 1.33)
Runny nose	48 (19)	30 (25)*	18 (14)	1.74 (0.89, 3.39)
Blocked nose	36 (15)	25 (21)**	11 (9)	2.36 (1.08, 5.17)

The groups were compared using chi-square tests and logistic regression, while adjusting for education.

N: Total number of samples; n: number of cases with the symptom; **P < 0.01 & *P < 0.05 (unadjusted chi-square tests); ^a^95 percent confidence interval.

### Fractional exhaled Nitric Oxide (FeNO)

A total of 114 female flower workers from Farm I & II participated in measurements of Fractional exhaled Nitric Oxide (FeNO), and valid results were obtained from 108 of these.

Among all participants FeNO ranged from 5 to166 ppb with a GM of 14 ppb (Table 6). Only two workers (1.8%) had FeNO concentration greater than 50 ppb, a level often used to indicate the presence of asthma [27]. The mean FeNO of those working inside and outside the greenhouses did not differ significantly, either among workers in Farm I and Farm II together or within each of the farms (Table 6). Workers in Farm I had significantly higher mean FeNO than in Farm II (GM = 15.8 ppb and 11.8 ppb; P = 0.009) respectively (Table 6). When excluding the two values above 50 ppb, the statistical differences between the groups did not change.

**Table 6:** **Mean FeNO level according to workstation (inside vs. outside greenhouse) and farm among the female flower farm workers**

**Farm ID**	**Workstation**	**N**	**AM**	**Range**	**Ns > 50 ppb**	**GM**	**GSD**
Farm I	IGH	26	16.5		0	15.3	1.47
	OGH	37	21.4		1	16.2	1.93
	Total^a^	63	19.4	5-166	1	15.8*	1.75
Farm II	IGH	23	17.2		1	12.8	1.95
	OGH	22	12.3		0	10.8	1.65
	Total^a^	45	14.8	5-107	1	11.8*	1.82
Total	IGH	49	16.8		1	14.1	1.72
	OGH	59	18.0		1	13.9	1.88
	Total	108	17.5	5-166	2	13.9	1.81

^a^Independent *t*-test; *P < 0.01 when comparing Farm I and II; Ns: Number of samples; IGH: Inside Greenhouse; OGH: Outside Greenhouse (packhouse).

## Discussion

Our study indicated that endotoxin exposure and FeNO levels were low while there were high prevalences of self-reported respiratory symptoms among the female workers in the flower farms. Greenhouse workers had significantly higher endotoxin exposure and reported more symptoms than workers outside greenhouses. However, after adjusting for education, only blocked nose remained significant. FeNO levels differed significantly between the farms but there was no difference comparing inside vs. outside greenhouse workers.

All the endotoxin samples, except one, were below the Dutch recommended health-based exposure limit value (90 EU/m^3^) [26]. Nevertheless, the mean endotoxin exposure was higher than reported for flower/ornamental growers inside European greenhouses (GM = 2.9; range 0.4-101.4 EU/m^3^) [28]. Studies among Dutch and Danish flower growers in greenhouses reported similar or slightly higher endotoxin exposure (GM = 27 & 44 EU/m^3^ respectively) [20,29]. In those studies they used inhalable dust samplers which have been reported to sample 1.5-4 times more dust by mass than the “total” dust samplers used in the present study [30]. Thus, it is difficult to directly compare the obtained results with other studies and with the Dutch limit value since the total dust samplers in our study presumably collected lower levels of endotoxins than were present in the inhalable dust fraction. A study among cucumber and tomato employees in greenhouses reported considerably higher endotoxin exposure (median = 320 EU/m^3^) which might be partly due to the larger leaf areas of these plants [9]. Greenhouse workers in Farm I had the highest endotoxin exposure of all the groups in both farms. Use of biological pest control method in Farm I might have contributed to the higher exposure, as this is the only apparent difference between the farms. One may speculate whether biological pest control combined with less use of pesticides could be related to this differences in exposure.

The day-to-day variance was higher than the between worker variance for the endotoxin exposure. One reason might be temporary relocation of workers to other greenhouses during spraying. However, we did not systematically register the relocation times. The fixed factors in the mixed-effects model only explains the between-worker variability in endotoxin exposure, which is logical since the individual worker did not change workstation (inside/outside greenhouse) or farm.

The greenhouse workers had higher prevalence for most symptoms than workers outside. However, when we adjust for education, only the prevalence of blocked nose, which is most likely a symptom of rhinitis, was significantly higher. This is similar to the findings in Croatia that female greenhouse workers had higher prevalence of respiratory symptoms than controls [2]. However, the prevalence of rhinitis in the Croatian study was higher (42%) than for blocked nose in the present study (21%). This difference may be explained by the use of different terms describing nose symptoms. The term rhinitis is broad, and more people may have reported this symptom than the more specific symptom blocked nose. Rhinitis is known to precede asthmatic conditions [31], which might mean that the workers inside greenhouses could be at risk of developing asthma. However, only surveys over a longer time can support this. The results in the present study are also partly consistent with our previous study [13], showing high prevalence of respiratory symptoms, including blocked nose. However, in that study a different control group was used. The low endotoxin exposure in the present study is not likely to explain the respiratory symptoms, and might rather be related to other components in the bioaerosol including fungi or pesticides [3,15]. Studies on greenhouse workers in Oman [32] and Turkey [33] reported prevalence of cough of 30% and 31%, respectively. The higher prevalence in these two studies than in the present study (19% of day/night time cough), might be explained by the higher age (78% over 30 years), cigarette smoking (19.8%) and longer work experience (73% over 7 years) of the workers in the Oman study. It might be possible that longer work experience with longer exposure time cause more respiratory health problems than we have been able to demonstrate.

This relatively high prevalence of shortness of breath, chest tightness and wheezing, even in the young worker population in our study, might be partly due to unknown factors such as reporting errors or living conditions. Over a quarter of the workers had domestic animals, which may also expose them to dust and allergens.

The mean FeNO in our study was GM = 13.9 ppb, which is considered to be low [34]. A study among endotoxin exposed female agricultural workers also found to be low FeNO (GM = 11.4 ppb) [35]. Previous studies of asthmatics have shown elevated FeNO levels [36,37]. Although we found low FeNO level in our study, it may not necessarily mean that these workers do not have any risk of asthma or other respiratory problems.

### Strength and limitation

Strengths of the present study are that objective measurements were done both for endotoxin exposure and FeNO. Moreover, the subgroups (inside vs. outside greenhouses) were comparable for most variables except education, which we controlled for in the analysis.

However, it is a weakness that we only measured endotoxin exposure during one summer (rainy and wet). This may not represent the winter (sunny and dry) exposure. A high fraction of the endotoxin samples had values below the limit of quantification at the laboratory. The high fraction of these low levels has presumably influenced the estimates of mean exposures and the fixed factors in the mixed-effects model, but presumably they did not affect the main findings. Pesticides and fertilizers of different types were widely used in the flower farms [13], but we were unable to measure these exposures in this study. There might be a healthy worker effect as most workers had short work experience due to high turnover. In addition, the administration may have picked the healthiest workers for examination. However, the administrative personnel was not likely to know all the details of the workers’ health.

## Conclusion

Greenhouse workers in flower plants had higher prevalence of blocked nose than those outside greenhouses, which may indicate the presence of rhinitis. Endotoxin exposure was low, but the levels were highest inside the greenhouses. There were few workers with objective signs of airway inflammation, but most workers had only been working in the greenhouses for two years. Further studies should be performed in the rose farms to evaluate the effect of longer employment and exposure time as well as to investigate possible occupational exposure to pesticides and other components in the bioaerosol.

## Abbreviations

FeNO: Fractional exhaled nitric oxide
GM: Geometric mean
BMRC: British medical research council
TB: Tuberculosis
PPB: Parts per billion
NO: Nitric oxide
LOQ: Limit of quantification
LOD: Level of detaction
EU/m^3^: Endotoxin unit per cubic meter
RPD: Respiratory protective devices
